# The Hot Vaginal Douche

**Published:** 1874-11

**Authors:** 


The Hot Vaginal Douche employed, by Dr. Emmet, (see page 473,) we have found of great service in our gynæcological cases. We have not, however, in the employment of it found occasion for the remarks of Dr. Emmet: "The only position in which the patient can receive any benefit from them is on the back, with the hips elevated, as described. She cannot administer them properly herself, and I know of no arrangement which can take the place of an intelligent nurse."

We find very satisfactory results from the use of the douche, as administered by the patient herself, sitting over a suitable receptacle and pumping the hot water from a bowl with an ordinary rubber syringe. It is only necessary to compress the labiæ with the fingers of the left hand, so as to offer an obstacle to the ready escape of the liquid and cause it to inflate the vagina fully. In this way the walls of the vagina and cervix uteri are kept bathed with hot water for any desired length of time.

---

The Address in Obstetrics. Delivered before the Medical Society of the State of Pennsylvania, May, 1874. By William B. Atkinson, M.D., etc., Physician to the Department of Obstetrics and Diseases of Women, Howard Hospital, Philadelphia. Philadelphia: 1874, pamphlet, pp. 43.

---

Tinnitus Aurium, or Noises in the Ears. By Laurence Turnbull, M.D., Physician to the Department of Diseases of the Eye and Ear, Howard Hospital. Philadelphia: J. B. Lippincott & Co., 1874, pamphlet, pp. 16.

---

On Deaf-Mutism and the Method of Educating the Deaf and Dumb. By Laurence Turnbull, M.D., Physician to the Department of Diseases of the Eye and Ear, Howard Hospital Philadelphia: Pamphlet, pp. 7.

